# Assessment of horse breeding and husbandry practices in southwest Ethiopia: Its implication to design breeding program

**DOI:** 10.1016/j.heliyon.2024.e39280

**Published:** 2024-10-18

**Authors:** Amine Mustefa, Aweke Engdawork, Seble Sinke

**Affiliations:** Ethiopian Biodiversity Institute, Addis Ababa, Ethiopia

**Keywords:** Culling, Draft-power, Gesha, Keffa, Selection, Sheka, Transport

## Abstract

Ethiopian horses are multipurpose horses that have socioeconomic significance for smallholder farmers. However, studies regarding their husbandry practices have received little attention. Thus, the current study was conducted to assess the horse husbandry practices in the southwest Ethiopia. Data were collected through semi-structured questionnaires from a total of 196 randomly selected respondents. The general linear model and frequency procedures of the Statistical Analysis System (SAS 9.0) were used to analyze the quantitative and qualitative data, while indices were calculated using Microsoft Excel 2016. Most of the respondents from the Telo district were illiterate, while the percentage of literate farmers was greater in the Gesha and Masha districts. The within-household flock structure and level of importance were dominated by cattle and sheep, followed by horses. The horses were used for transport, draft power, and breeding purposes. In terms of shelters, the horses in the Telo district stayed in shelters, while the horses in the Masha district stayed in forests during both the dry and wet seasons. However, the majority of the horses in the Gesha district were sheltered during the wet season, while they were left to stay in the forest during the dry season. The respondents provided supplementary feed and water to their horses while the water point was located within a kilometer distance. Government and private veterinary shops were the primary sources of veterinary services. Farmers sold their horses at the local market. Castration was performed to minimize aggressiveness. Body size, conformation, and temperament were used as sire selection criteria. An increasing trend in horse population size due to increased farmer interest was observed. In the studied areas, horses were found to be highly important to the livelihood of the farmers. However, horse husbandry practices were led by a considerably greater number of illiterate farmers. Moreover, horse management activities, including the housing of horses, need more attention. Therefore, successive awareness-raising campaigns, including the introduction of formal schooling, are recommended to improve the horse breeding and husbandry practices. Moreover, breeding programs with the aim of genetic improvement and conservation need to be designed to optimize the sustainable utilization of the horses.

## 1 Introduction

Ethiopian horses, reportedly 2.14 million head [1], contribute significantly to the transportation and agricultural production of Ethiopia. Horses play a vital role in the transportation of humans as well as several agricultural products. In the rural areas of the country where motor vehicles have not yet been introduced, horses are the primary and main source of transport. In most urban and peri-urban areas of the country, it is also common to observe carts pulled with horses [2]. Moreover, the majority of the rural people who live in the highland areas of the Keffa and Sheka zones use horses permanently to transport agricultural products to the market as well as other materials from the market [3]. [2] also reported the use of horses as a draft power where several land cultivation activities were practiced using horse powers.

In addition to their significance in the transport and agriculture sector, the majority of Ethiopian horses, especially those found in the highlands of the Keffa and Sheka zones, are also used for various cultural and social events and racing shows [3]. According to Ref. [3], horses are highly valued and culturally respected domestic animals all over the country in general and specifically in the Keffa and Sheka zones.

Although horses are multifunctional animals, research and development activities that are planned to study and improve their husbandry practices have received little attention. To date, few phenotypic and genetic characterization studies have been conducted to quantify the relationships among Ethiopian horse breeds [4]. Accordingly, eight horse breeds were reported to exist in the country [3] and are further categorized into three clusters [5]. However, studies regarding horse management, horse production, farmers’ breeding practices, breeding objectives, and selection and culling criteria have not yet been carried out. Studying these management practices and breeding practices is important for designing breed- and area-specific breeding and conservation programs.

The current study targeted the Keffa horse breed, which is reported to be found in the highland areas of the Keffa and Sheka zones of the southwestern Ethiopia region [3]. The existence of the Gesha horse breed in the Gesha district of the Keffa zone was also reported by Ref. [4]. Keffa horses are forest-type horses known for their large body size, coarse body, well-sprung ribs, long mane, and high thorax girth among the other Ethiopian horse breeds [3]. Gesha horses, which are typical saddle horses, are the tallest horses in Ethiopia and are also known for their aggressive behavior and red body color [4]. Therefore, in addition to their phenotypic study, assessments of their management and breeding practices as well as farmers' selection and culling criteria are required to design breeding and conservation programs. Thus, the current study was conducted to assess horse management and breeding practices as well as farmers’ selection and culling criteria in the Telo, Gesha, and Masha districts of the Keffa and Sheka zones in southwest Ethiopia.

## 2 Materials and methods

### 2.1 Study areas

**Fig. 1:**
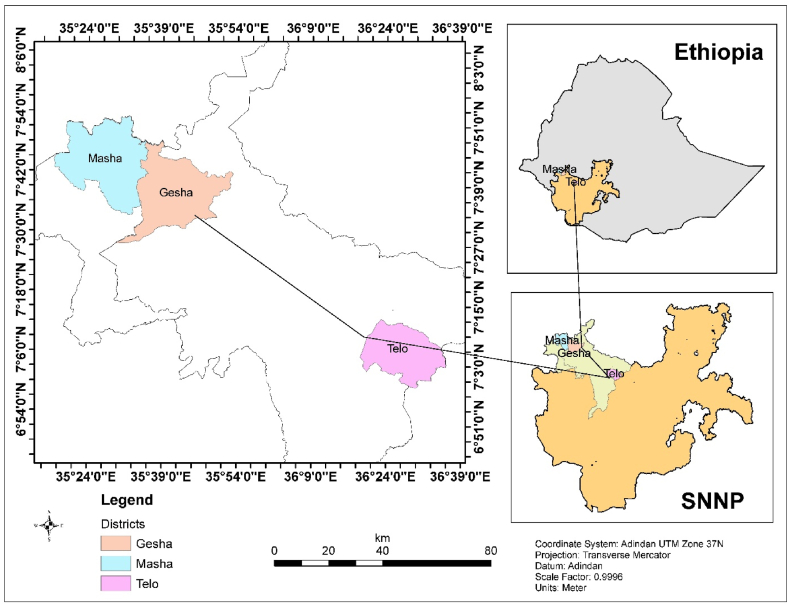
Map of the study areas [4].

The current study was carried out in two zones, i.e., the Keffa and the Sheka zones of the southwest Ethiopia region (Fig. 1). Three districts were selected, namely, the Telo, Gesha, and Masha districts. The Telo district has an altitude range of 2436–2451 m above sea level (m.a.s.l.), a temperature range of 17–25 °C, and an average rainfall of 1278 mm [6]. The Gesha district has an altitude range of 2501–3000 m.a.s.l., a temperature range of 15.1–20 °C, and a rainfall range of 2001–2200 mm [7]. The Masha district has an altitude range of 1700–3000 m.a.s.l., an average temperature of 16.7 °C, and an average rainfall of 2192 mm [8]. According to the [1], the horse population sizes in the Keffa and Sheka zones were 59,343 and 14,721, respectively.

### 2.2 Selection strategy

Information regarding the distribution areas of the horse breeds was obtained using secondary information. The Telo and Gesha districts were selected from the Keffa zone, while the Masha district was selected from the Sheka zone. The Gesha and Masha districts were selected to represent the Gesha and Keffa horse breed distribution areas, respectively, while the Telo district was selected randomly from the Keffa horse breed distribution area. Two sampling sites (‘***Kebeles’*** or the smallest administrative units) were randomly selected from each district. A total of 196 households that reared horses were randomly selected using the [9] formula (1.96)2 (0.85) ∗(0.15)/0.052 = 196. Accordingly, 62 households from the Telo and Masha districts and 72 households from the Gesha district were selected.

### 2.3 Data collection

Data collection was carried out according to the [10] guidelines for livestock production system characterization. The Amharic version of the semistructured questionnaire was prepared by the Animal Biodiversity Directorate (ABD) of the Ethiopian Biodiversity Institute (EBI) [11], which is the country's directorate responsible for the characterization of indigenous animal genetic resources and their production system. The questionnaire was pretested prior to the final data collection. Accordingly, several datasets, including household characteristics (education status, age, horse ownership, and family size), quantitative traits (overall livestock and horse flock structures), qualitative traits (housing, feeding, watering, selling, castration, veterinary care, sire source, population size trend and reason, origin of the horses), and ordered traits (preferred livestock species, reason for keeping horses, selection and culling criteria), were collected.

### 2.4 Data analysis

The Microsoft Office Excel worksheet was used to enter and manage the data, and the overall data analysis was carried out using the Statistical Analysis System (SAS) software 9.0 [12]. The UNIVARIATE procedure for the data normality test, the frequency procedure (chi-square test) for qualitative data analysis, and the general linear model (GLM) procedure for quantitative data analysis were used. The following analysis model was used to analyze the data. Y_i_ = μ + D_i_ + e_i,_ where Y_i_ is an observation, μ is the overall mean, D_i_ is the fixed effect of district (i = Telo, Gesha, Masha) and e_i_ is the random error. Some sex-sensitive traits were analyzed separately for each sex by fitting district as a class variable. Means (LSM) were separated using the adjusted Tukey‒Kramer test at the 5 % level of significance. The indices were calculated using the following formula. Index = sum of [3∗1st ranked trait+ 2∗2nd ranked trait + 1∗3rd ranked trait] of a particular variable divided by the overall sum of all variables within each district [13]. The indices were calculated using Microsoft Excel 2016.

## 3 Results

### 3.1 Household characteristics

The percentage and chi-square values of the household characteristics along with the probability of significance of each trait under the three studied districts are presented in Table 1. The overall results show a relatively higher level of educated respondents, where the majority of the respondents fall under the fourth group. District significantly affects all traits. Accordingly, most of the respondents from the Telo district were found to be illiterate, while lower levels of illiteracy were found in the Gesha district. The education status of the respondents from Masha District fell between that of the respondents from the other two districts. The age of the respondents was significantly affected by district, where the majority of the respondents from the Gesha and Masha districts were distributed in the medium age category (2nd and 3rd age group), while relatively higher percentages of old and young respondents were observed in the Telo district. Horse ownership within each family was also significantly affected by the district where the male head was the horse owner in the majority of the Telo and Masha districts, while the whole family was the owner of the horses in the Gesha district.

**Table 1:** Age and education level of the households. Percentage values.

Household characteristics	Telo	Gesha	Masha	Overall	*ꭓ* 2 value	Prob.
Education					19.0	∗
Illiterate	37.1	8.3	22.6	22.0		
Read + write	8.1	12.5	9.7	10.2		
Grades 1–4	16.1	18.1	12.9	15.8		
Grades 5–8	21.0	41.7	30.6	31.6		
Grades 9–12	17.7	19.4	24.2	20.4		
Age (years)					22.5	∗∗
15–30	17.7	5.6	12.9	11.7		
31–40	19.4	37.5	27.4	28.6		
41–50	21.0	37.5	40.3	33.2		
51–60	24.2	8.3	14.5	15.3		
61–70	17.7	11.1	4.9	11.2		
Horse ownership					93.0	∗∗∗
Male head	54.8	2.8	51.6	34.7		
Female head	0.0	5.6	19.4	8.2		
Spouses together	0.0	20.8	0.0	7.6		
Sons	0.0	0.0	3.2	1.0		
The whole family	45.2	70.8	25.8	48.5		

Prob. Probability, ∗p < 0.05, ∗∗p < 0.01, ∗∗∗p < 0.0001.

The effect of district on male and female family size is also presented in Table 2. District differences did not significantly affect female family size but did affect male family size. Accordingly, the Telo district was observed to have the greatest male family size, while the other two districts had comparable family sizes.

**Table 2:** Family size of each household per district (LSM ± SE).

Family size	Telo	Gesha	Masha	Overall	Sig.
Males	5.1 ± 0.23^a^	4.1 ± 0.22^b^	3.9 ± 0.23^b^	4.3 ± 0.14	∗∗
Females	4.2 ± 0.29	4.2 ± 0.27	3.4 ± 0.29	3.9 ± 0.16	NS

∗p < 0.05, ∗∗p < 0.01, ∗∗∗p < 0.0001.

### 3.2 Flock structure and preference

The means (LSM ± SE) and pairwise comparisons of the livestock flock structure of the three districts are presented in Table 3. The numbers of cattle, sheep, goats, and bee hives were significantly affected by the district of the respondents, while the numbers of chickens, horses, and mules per household were comparable across the studied districts. The respondents from the Gesha district possessed the most cattle and goats, while those from the Masha district possessed the most bee hives. The results also showed that the respondents from the three districts did not possess camels or donkeys in their household.

**Table 3:** Livestock flock structure per household (LSM ± SE).

Flock structure	Telo	Gesha	Masha	Overall	Sig.
Cattle	9.5 ± 0.90^b^	15.3 ± 0.84^a^	9.2 ± 0.90^b^	11.5 ± 0.54	∗∗∗
Sheep	8.0 ± 0.64^a^	8.6 ± 0.60^a^	4.9 ± 0.64^b^	7.3 ± 0.38	∗∗∗
Goats	1.6 ± 0.36^b^	4.1 ± 0.34^a^	0.8 ± 0.36^b^	2.3 ± 0.23	∗∗∗
Chicken	7.4 ± 0.82	8.4 ± 0.76	7.5 ± 0.82	7.8 ± 0.46	NS
Horses	2.6 ± 0.18	2.9 ± 0.17	2.3 ± 0.18	2.6 ± 0.10	NS
Mules	0.1 ± 0.05	0.2 ± 0.04	0.1 ± 0.05	0.14 ± 0.03	NS
Bee hives	1.3 ± 1.05^c^	3.6 ± 0.98^b^	10.8 ± 1.05^a^	5.1 ± 0.65	∗∗∗
Camel	0	0	0	0	NA
Donkey	0	0	0	0	NA

NS: not significant, NA: not available, ∗p < 0.05, ∗∗p < 0.01, ∗∗∗p < 0.0001.

The index values of the importance of the different livestock species to the farmers in the three districts are presented in Table 4. Accordingly, cattle, sheep, and horses were the three most important livestock species in the Telo and Masha districts, while the respondents from the Gesha district preferred goats over horses.

**Table 4:** Importance of livestock species to the farmers in the study areas (index values).

Districts	Cattle	Sheep	Goats	Chicken	Horses
Telo	0.45	0.27	0.02	0.11	0.15
Gesha	0.47	0.28	0.10	0.07	0.08
Masha	0.50	0.27	0.03	0.06	0.14
Overall	0.48	0.27	0.05	0.08	0.12

**Table 5:** Flock structure of horses per household (LSM ± SE).

Districts	Mares	Stallions	Young females	Young males
Sig.	NS	∗	NS	∗∗∗
Telo	1.3 ± 0.11	0.6 ± 0.08^b^	0.3 ± 0.07	0.3 ± 0.06^a^
Gesha	1.1 ± 0.10	0.9 ± 0.07^a^	0.4 ± 0.06	0.5 ± 0.06^a^
Masha	1.2 ± 0.11	0.8 ± 0.08^ab^	0.2 ± 0.07	0.06 ± 0.06^b^
Overall	1.2 ± 0.06	0.8 ± 0.04	0.3 ± 0.04	0.3 ± 0.04

∗p < 0.05, ∗∗p < 0.01, ∗∗∗p < 0.0001.

The means (LSM ± SE) and pairwise comparisons of the horse flock structure of the three districts are presented in Table 4. The number of adult and young male horses was significantly affected by breed, while the number of adult and young female horses was comparable across the three districts. The respondents from the Gesha district possessed a relatively greater number of adult and young male horses than did those from the other two districts.

### 3.3 Objectives of horse production

The index value results showing the purpose of keeping male and female horses in the three districts are presented in Table 6. Overall, the respondents kept male horses for transport and draft power; however, the female horses were mainly kept for breeding, followed by transport and draft power. Compared with male horses, female horses were better sources of income because they were not used in cultural ceremonies. Comparing districts, male horses in the Telo and Gesha districts were mainly kept for transportation purposes, while their counterparts from the Masha district were primarily used for draft power. On the other hand, the female horses of the Telo and Masha districts were mainly kept for breeding, while their counterparts from the Gesha district were jointly used for transport and draft power.

**Table 6:** Farmers’ reasons for keeping male and female horses (index values).

Use	Male horses				Female horses			
	Telo	Gesha	Masha	Overall	Telo	Gesha	Masha	Overall
Transport	0.51	0.40	0.32	**0.41**	0.14	0.31	0.24	**0.23**
Draft power	0.32	0.28	0.43	**0.34**	0.18	0.30	0.15	**0.21**
Breeding	0.01	0.08	0.04	**0.04**	0.36	0.24	0.36	**0.32**
Wealthy status	0.05	0.11	0.02	**0.06**	0.12	0.04	0.05	**0.07**
Cultural ceremony	0.04	0.07	0.03	**0.05**	0	0	0	**0**
Income source	0.07	0.06	0.16	**0.10**	0.20	0.11	0.20	**0.17**

### 3.4 Horse management practices

The percentage and chi-square values of each management practice along with the probability of significance of each trait under the three studied districts are presented in Table 7. The majority of the horses stay in the forest during the dry season, while the farmers purposively made houses for their horses during the wet season. However, this response was significantly affected by the district of the respondents. Horses from Masha District stay in the forest during both seasons, while the horses from Telo District stay in a shelter purposively made for them and with other animals during the wet and dry seasons, respectively. The majority of the horses in the Gesha district were sheltered by other animals during the wet season, while they were left to stay in the forest in the dry season.

**Table 7:** Horse management (percentage values).

Management practices	Telo	Gesha	Masha	Overall	*ꭓ* 2 value	Prob.
Housing in dry season					91.3	∗∗∗
Purposively made for horses only	0.0	8.3	0.0	3.1		
With other animals	40.3	40.3	17.7	33.2		
Temporary shelter	38.7	0.0	0.0	12.2		
In the forest	21.0	51.4	82.3	51.5		
Housing in wet season					95.5	∗∗∗
Purposively made for horses only	66.1	26.4	16.1	35.7		
With other animals	24.2	43.0	12.9	27.5		
Temporary shelter	9.7	0.0	0.0	3.1		
In the forest	0.0	30.6	71.0	33.7		
Do you give supplementary feed?					34.5	∗∗∗
Yes	85.5	97.2	58.1	81.1		
No	14.5	2.8	41.9	18.9		
Do you give water?					2.9	NS
Yes	95.2	87.5	87.1	89.8		
No	4.8	12.5	12.9	10.2		
Distance to water point in dry season					85.6	∗∗∗
In compound	8.1	9.7	46.8	20.9		
<1 km	79.0	36.1	53.2	55.1		
1–5 km	12.9	54.2	0.0	24.0		
Distance to water point in wet season					45.1	∗∗∗
In compound	45.2	8.3	46.8	32.1		
<1 km	25.8	69.5	50.0	49.5		
1–5 km	29.0	22.2	3.2	18.4		
Where do you get veterinary service?					177.5	∗∗∗
Government	77.4	2.8	30.6	35.2		
Private veterinary clinic	0.0	11.1	0.0	4.1		
Private veterinary shop	22.6	69.4	0.0	32.6		
All	0.0	16.7	69.4	28.1		
Where do you sell your horses?					21.6	∗∗
At local market	95.2	90.3	79.0	88.3		
To neighbor farmers	0.0	0.0	14.5	4.6		
Both	4.8	9.7	6.5	7.1		

Prob. Probability, NS: not significant, ∗p < 0.05, ∗∗p < 0.01, ∗∗∗p < 0.0001.

The majority of the respondents provided supplementary feed and water, while the water point was found within a kilometer during both the dry and wet seasons. Government and private veterinary shops were the main sources of veterinary services. However, this was significantly affected by the district of the respondents, where the majority of the respondents from Telo district received veterinary services primarily from the government, while private veterinary shops were the primary sources of veterinary services in Gesha district. On the other hand, both of them were sources of veterinary services in Masha district. The majority of the respondents sold their horses at the local market, while a significant number of respondents from Masha district sold their horses to neighboring farmers.

### 3.5 Breeding practices

The percentage and chi-square values of the farmers’ breeding practices along with the probability of significance of each trait under the three studied districts are presented in Table 8. Accordingly, all of the respondents had practiced castration, and attaining a better temperament was the reason for the majority of the respondents. Communal sires were used by the majority of the respondents, while significantly more respondents from the Gesha district used sires from their own flocks.

**Table 8:** Breeding practices of the farmers (percentage values).

Breeding practices	Telo	Gesha	Masha	Overall	*ꭓ* 2 value	Prob.
Do you practice castration?					NA	NA
Yes	100	100	100	100		
No	0.0	0.0	0.0	0.0		
Reason for castration					15.2	∗∗
For better draught power	12.9	7.0	6.5	8.7		
For better temperament	80.6	59.7	69.3	69.4		
For both	6.5	33.3	24.2	21.9		
Do you practice culling?					NA	NA
Yes	100	100	100	100		
No	0.0	0.0	0.0	0.0		
Where do you get sire from?					33.9	∗∗∗
Own flock sire	25.8	47.2	24.2	33.2		
Borrowed sire	0.0	20.8	8.1	10.2		
Communal sire	74.2	32.0	67.7	56.6		

Prob. Probability, NA: not available, ∗p < 0.05, ∗∗p < 0.01, ∗∗∗p < 0.0001.

### 3.6 Horse culling criteria

All of the interviewed respondents had practiced culling of their unwanted horses from their flocks (Table 8). The index value results showing farmers’ culling criteria for male and female horses in the three districts are presented in Table 9. Temperament, body size, and conformation were the top three culling criteria for male horses, while the culling criteria for female horses were breeding, body size, and temperament. Age, aggressiveness, small body size, bad body conformation for males and low reproductive ability, small body size, and aggressiveness for females are the top three culling criteria.

**Table 9:** Farmers’ criteria for culling male and female horses (index values).

Culling criteria	Male horses				Female horses			
	Telo	Gesha	Masha	Overall	Telo	Gesha	Masha	Overall
Body size	0.30	0.21	0.17	**0.23**	0.19	0.24	0.17	**0.20**
Conformation	0.15	0.24	0.21	**0.20**	0.06	0.12	0.14	**0.11**
Body color	0.22	0.05	0	**0.09**	0.02	0.02	0.01	**0.02**
Temperament	0.23	0.19	0.35	**0.26**	0.09	0.16	0.18	**0.14**
Health issue	0.01	0.20	0.10	**0.10**	0.14	0.14	0.10	**0.13**
Age	0.09	0.11	0.17	**0.12**	0.12	0.07	0.10	**0.09**
Breeding	0	0	0	**0**	0.38	0.25	0.30	**0.31**

In terms of district, body size, temperament, and body color in the Telo district; conformation, body size, and health issues in the Gesha district; and temperament, conformation, body size and age in the Masha district were the top culling criteria for male horses. Similarly, breeding, body size, and health issues in the Telo district; breeding, body size, and temperament in the Gesha district; and breeding, temperament, and body size in the Masha district were the top culling criteria for female horses.

### 3.7 Stallion selection criteria

The index value results showing farmers’ sire selection criteria in the three districts are presented in Table 10. Accordingly, body size, conformation, and temperament were the three most common sire selection criteria. Among the districts, body size, conformation, and temperament in the Telo district; conformation, body size and temperament in the Gesha district; and temperament, conformation and body size in the Masha district were the top three sire selection criteria.

**Table 10:** Farmers’ sire selection criteria (index values).

District	Body size	Conformation/shape	Body color	Temperament
Telo	0.36	0.23	0.18	0.23
Gesha	0.30	0.37	0.12	0.21
Masha	0.25	0.31	0.09	0.35
Overall	0.30	0.30	0.13	0.27

### 3.8 Population size trend

The percentage and chi-square values of some population size-related parameters along with the probability of significance of each trait under the three studied districts are presented in Table 11. The majority of the participants exhibited an increasing trend in their horse populations, while a significant number of respondents from the Gesha and Masha districts exhibited a decreasing trend in population size. The overall reason for the increase in horse population size was the increase in farmers’ interest in their horses. Moreover, the majority of the respondents considered their own district to be the origin of their indigenous horse breeds.

**Table 11:** Population size-related parameters (percentage values).

Parameters	Telo	Gesha	Masha	Overall	*ꭓ* 2 value	Prob.
Population size trend of the horses					59.6	∗∗∗
Increasing	92.0	65.3	40.3	65.8		
Decreasing	3.2	34.7	29.0	23.0		
Stable	4.8	0.0	30.7	11.2		
Reason for the population size increase					27.9	∗∗∗
Due to the increased farmers' interest	77.4	34.7	40.3	50.0		
Due to the easily available breeding	14.5	41.7	35.5	31.1		
Due to its better draught power	8.1	23.6	24.2	18.9		
Origin of the horses?					1.6	NS
Own district	85.5	91.7	85.5	87.8		
Neighboring district	14.5	8.3	14.5	12.2		

Prob. Probability, NS: not significant, ∗p < 0.05, ∗∗p < 0.01, ∗∗∗p < 0.0001.

## 4 Discussions

The level of education may be directly related to the level of management, where more educated farmers pay special attention to the management of their animals and agricultural activities [14]. Such special attention to their animals may arise from the knowledge they received from education. Moreover, educated farmers can accept and practice new production and conservation technologies [15]. Therefore, introducing and strengthening education in rural communities is recommended to improve the overall production and conservation of specific animals and agriculture in general [2]. However, in addition to regular education, successive awareness-raising campaigns are required to minimize the migration of educated people to urban areas of the country, as the greater the number of people educated, the greater the chance of migration to urban areas, which in turn has a negative impact on agricultural activities. This was supported by Ref. [2], who reported a greater impact of primary and secondary school than other higher education levels on agricultural development.

The age of the respondents might also affect management activities, as young and middle-aged respondents might have the strength to follow up with their animals. However, this might also affect management practices, as young farmers may pay less attention to their animals, as they might be attracted to more income-oriented agricultural activities. Therefore, providing successive training and awareness-raising campaigns is recommended, especially for young and aged groups. With respect to horse ownership, a specific horse owner within a family might be important for the management of the horses, as the indicated person takes all the responsibilities thinking that no one is responsible for the horses except him. However, when the whole family becomes the horse owners, they might love the horses and manage them better than a single owner.

Family size can be directly correlated with the horse management activities of the household, where there is a greater chance of better horse management in a larger family than in a smaller one. Accordingly, the large family size observed in the current study can be considered a boost to the animal and agricultural management activities of the areas. The family size in the current study was greater than that in Ref. [2] in the Awi zone and that in Ref. [16] in the Metema district.

The flock structure results have indirect relationships with the management priorities given to the horses. The observation of the greatest number of cattle per household in Gesha district might decrease the amount of attention given to their horses. On the other hand, horses from the Telo and Masha districts might receive more attention than their counterparts from the Gesha district. This was also supported by the results of Table 4, where horses in Gesha district were ranked fourth in terms of importance following cattle, sheep, and goats. In line with these results, horses in the Telo and Masha districts were ranked 3rd after cattle and sheep. In contrast to the current results [2], reported the dominance of horse number over other livestock species in the Ankesha Guagusa district of the Awi zone; however, the overall horse number was lower than that in the current study. The zero number of camels and donkeys observed in the households of the three districts shows the significance of horses in covering all transportation activities. The within-horse flock structure results shown in Table 5 might be due to the preference of male horses in the Gesha district over the other districts. The male horses of Gesha districts (especially the Gesha stallions) were reported to be typical riding horses and known for their high speed [4].

Comparable results regarding the purpose of keeping horses were reported by Ref. [2] in the Awi zone, who described the multiple uses of horses, including riding and plowing. These results were also in line with reports of [17] in the mid-rift valley of Ethiopia and [18] in the Meskan district, who described the multiple purposes of horses in a family.

The majority of the horses from Masha District stay in the forest most of their time, which might give them the characteristics of feral horses. These horses spent most of their time in the Keffa and Sheka forests, which are among the largest forest areas in the country. Their aggressive behavior might be due to their extended stay in the forest area, which is out of human control. This was also supported by the results of Table 9, where the respondents from Masha district put temperament as their top culling criterion where aggressive horses were not tolerated. Similarly, this was also supported by the feed supplementation results, where almost half of the respondents from Masha district did not supplement their horses, as their horses received ample feed in the forest. In contrast to the current findings, horses in the Awi zone were reported to stay within the family house [2].

There were more indoor horses in the Telo district than other horses. Such management activities improve the temperament of horses, as they spend more time interacting with humans. This finding was also supported by the results shown in Table 9, where the respondents from the Telo district had a temperament second highest after the body size trait when their culling criteria were met. The housing of the horses from Gesha district was found to be season dependent: they were more likely to be housed in the wet season than in the dry season. This was directly related to the rain pattern in which farmers shelter their horses to protect them from rain while allowing them to stay in the Keffa forest during the dry season to fulfill the horses’ feed requirements.

The distance to the water point was observed to be within a kilometer in most cases, while the majority of the horses from the Gesha district had to walk further to find water during the dry period. Therefore, building water points in the Gesha district is recommended to support horse production and conservation in the area.

Several critical issues affecting the sustainable utilization of the horse populations of the southwest Ethiopia region have been identified including the observed higher percentage of illiterate animal keepers, and the observed unimproved horse management activities regarding to housing, feeding, marketing, and health care. Therefore, practical strategies need to be developed and implemented to achieve economic, social, and cultural goals of the horse keepers. Accordingly, Community Based Breeding Programs (CBBP) need to be designed and implemented in a participatory approach both for conservation and genetic improvement of the horse populations. Several actors need to participate in the development and implantation of the CBBPs including policy makers, science communities, and practitioners. The policy makers can play a crucial role in developing, budgeting, and monitoring and evaluating of appropriate policy and legal framework taking sustainable utilization into account. The science communities i.e. universities and research centers, can play a pivotal role in undertaking further research, modeling suitable approaches, predicting potential outcomes, synthesizing knowledge through the use of innovation and technology. They are also expected to undertake capacity building activities including training and mentoring the horse owners. The practitioners can also directly implement the breeding programs on the ground. The practitioners are expected to translate policy and scientific knowledge into tangible results.

## 5 Conclusion

In the studied areas, horses were found to be highly important to the livelihood of the farmers. However, horse husbandry practices were led by a considerably greater number of illiterate farmers. Moreover, horse management activities, including the housing of horses, need more attention. Therefore, successive awareness-raising campaigns, including the introduction of formal schooling, are recommended to improve the horse breeding and husbandry practices. Moreover, breeding programs with the aim of genetic improvement and conservation need to be designed to optimize the sustainable utilization of the horses.

## CRediT authorship contribution statement

**Amine Mustefa:** Writing – review & editing, Writing – original draft, Methodology, Investigation, Funding acquisition, Formal analysis, Data curation, Conceptualization. **Aweke Engdawork:** Writing – review & editing, Methodology, Investigation, Data curation, Conceptualization. **Seble Sinke:** Writing – review & editing, Methodology, Investigation, Data curation, Conceptualization.

## Data availability statement

The data can be available from the corresponding author up on a reasonable request.

## Additional information

No additional information is available for this paper.

## Declaration of competing interest

The authors declare that they have no known competing financial interests or personal relationships that could have appeared to influence the work reported in this paper.
